# Mapping educational needs in bioinformatics in Brazil: adapting ISCB 3.0 competencies to a regional context

**DOI:** 10.1093/bioadv/vbaf311

**Published:** 2025-12-02

**Authors:** Bernardo Velozo, Clara Carvalho, Rayssa Feitosa, Lucas Aleixo Leal Pedroza, Emerson Danzer, Sandy Ingrid Aguiar Alves, Maira Neves, Bibiana Fam

**Affiliations:** Graduate Program in Biochemistry, Department of Biochemistry, Institute of Chemistry, Federal University of Rio de Janeiro, Rio de Janeiro, Brazil; Interunit Graduate Program in Bioinformatics, University of São Paulo, São Paulo, Brazil; Genetics and Genome Biology Department, The Hospital for Sick Children, Toronto, ON, M5G 1X8, Canada; Graduate Program in Biology Applied to Health, Keizo Asami Institute, Federal University of Pernambuco, Recife, Brazil; Interunit Graduate Program in Bioinformatics, Federal University of Minas Gerais, Belo Horizonte, Minas Gerais, Brazil; Graduate Program in Biology of Infectious and Parasitic Agents, Federal University of Pará, Belém, Pará, Brazil; Interunit Graduate Program in Bioinformatics, University of São Paulo, São Paulo, Brazil; Laboratory of Genomic Medicine, Center for Experimental Research, Hospital de Clínicas de Porto Alegre, Porto Alegre, RS, Brazil

## Abstract

**Motivation:**

Bioinformatics drives modern biological discovery, and Brazil has become an important contributor to genomics and computational biology. However, bioinformatics education across the country struggles to meet diverse regional and professional demands. To respond to these challenges, the Regional Student Group of Brazil created an Educational Committee in 2019 to expand Portuguese-language resources and evaluate national training needs. Here, we apply the Core Competency 3.0 framework to establish a seven-domain training model spanning foundational biological, statistical, and computational skills, ethical principles, applied bioinformatics practices, communication abilities, and continuous professional development.

**Results:**

A nationwide survey of 375 respondents from more than 21 Brazilian states revealed pronounced geographic and career-based disparities in bioinformatics training. Individuals who primarily use bioinformatics tools, largely students, showed strong interest in phylogenetics and evolutionary analyses, while those focused on software and tool development prioritized computational methods. These findings demonstrate how educational needs differ across profiles and regions, emphasizing the importance of localized strategies to address Brazil’s heterogeneous training landscape. Unlike broad competency frameworks, this data-driven approach identifies specific gaps and areas of high demand.

**Availability and implementation:**

By integrating these insights, the Regional Student Group of Brazil proposes an equitable and scalable education model that supports curriculum development and helps strengthen training in regions with limited opportunities, offering a framework adaptable to global scientific communities facing similar socioeconomic challenges.

## 1 Introduction

Bioinformatics and genomics in Brazil gained substantial momentum following the landmark sequencing of the citrus pathogen *Xylella fastidiosa*, a pioneering achievement for Brazil’s capacity for large-scale genomics and a landmark achievement of Bioinformatics in Brazil (Simpson *et al.* 2000). Over the subsequent two decades, these fields have made significant advancements (Rocha *et al.* 2022). However, despite these developments, bioinformatics training curricula require revision to align with global standards and practices (Emery and Morgan 2017). Integrating knowledge of bioinformatics’ strengths and limitations is essential for driving high-throughput data-driven research (Pucker *et al.* 2019). This necessity has fostered a specialized framework that accelerates scientific discovery and demands diverse skills, underpinned by bioinformatics core competencies (Mulder *et al.* 2018). To address gaps in bioinformatics education within university curricula and understand community requirements, the International Society for Computational Biology’s Community of Special Interest in Education (ISCB EduCOSI) developed documented training resources and tutorials. These establish core competencies as tools to support bioinformatics professionals and aid in creating field-relevant courses and curricula. (Kilpatrick *et al.* 2022). The transition from Core Competencies 2.0 (Mulder *et al.* 2018) to 3.0 (Brooksbank *et al.* 2024) reflects the field’s continuous evolution and aligns with its educational needs.

The conceptualization of the ISCB Core Competencies began in 2012, initially informed by surveys distributed to 50 ISCB members and 79 representatives from the European Molecular Biology Network (EMBnet) community (Welch *et al.* 2016). Since this initial effort, three iterations of the Core Competencies have been developed, each evolving to address continuous advancements in bioinformatics. These competencies now encompass a comprehensive range of knowledge, skills, and attitudes required for bioinformatics-related professions (Brooksbank *et al.* 2024), addressing critical aspects of professional development, including life sciences expertise, data analysis, ethical considerations, and effective communication (Welch *et al.* 2014).

However, bioinformatics remains unevenly implemented across Brazilian higher education institutions. Due to its relative novelty and limited familiarity, integration into academic curricula is sparse, confined to specialized centers. Some key obstacles to incorporating bioinformatics into undergraduate programs include outdated computing infrastructure, language barriers, and insufficient investment in technology (Dorn *et al.* 2021, Silva *et al.* 2021).

In this context, the ISCB Regional Student Group of Brazil (RSG-Brazil) has operated within the Brazilian academic landscape [2009–2025 (ongoing)] as a student organization dedicated to advancing bioinformatics through education, development, and outreach. Our mission is to democratize access to bioinformatics across Brazil through collaborative networks, leadership opportunities, training programs, and mentorship for students and early-career researchers. To advance this mission, RSG-Brazil established its Educational Committee in 2019 with dual objectives: evaluating nationwide training needs and developing Portuguese-language educational resources. We adopted the ISCB EduCOSI framework and its Core Competencies, addressing specialized knowledge and skill requirements from biological sciences to coding and applied statistics. Competency refinement and training model development incorporated contributions from members across Brazil’s regions, who leveraged their training experiences to provide critical perspectives, ultimately enriching this work.

As global demand for qualified bioinformaticians intensifies, RSG-Brazil collaborates with the Brazilian Association of Bioinformatics and Computational Biology (AB³C) and ISCB to contribute to international discourse on identifying needs, targeting audiences, and designing effective bioinformatics training programs. This work describes RSG-Brazil’s application of ISCB Core Competencies to assess educational demands and researcher profiles across Brazil. We specifically map the diversity of topics required in bioinformatics training, a central objective of this study. To this end, we implemented the ISCB Core Competencies training model to evaluate technical priorities identified by the Brazilian community. This framework, structured around seven core domains, informs RSG-Brazil’s state-specific strategies for advancing bioinformatics education nationwide.

## 2 Methods

### 2.1 Assembly of an educational committee

The Educational Committee of RSG-Brazil comprises volunteer members with diverse academic backgrounds. Established in 2019, the Committee initially analyzed and reclassified Core Competencies 2.0 into distinct educational domains (Table 1, available as supplementary data at *Bioinformatics Advances* online), defined as: (i) Knowledge in Biology; (ii) Statistical Methods; (iii) Computing Knowledge and Interdisciplinary Programming; (iv) Ethical Implications and Repercussions of Bioinformatics in Society; and (v) Uses of Bioinformatics and Communication. Reconvened in July 2023 with an expanded scope, the Committee leveraged prior work to analyze Core Competencies 3.0, reviewing newly defined Knowledge, Skills, and Attitudes (KSAs) per domain while integrating categories from version 2.0. The methodology for mapping Core Competencies 3.0 and its KSAs across seven domain topics is detailed in the following section.

**Table 1:** Cross-framework alignment of refined bioinformatics education domains with ISCB Core Competencies (v2.0-v3.0) and Bloom’s Taxonomy categories.

Domain topics in bioinformatics education and training	ISCB—core competencies 2.0	Bloom’s taxonomy
This work	Brooksbank *et al.* (2024)	Anderson and Krathwohl (2001)
Knowledge in Biology	A3	Cognitive
Methods in Statistics and Data Science	D3	
Knowledge of Computing and Interdisciplinary Programming in the area	G3, H3, I3	
Ethical Implications and Repercussions of Bioinformatics in Society	E3, J3	
Uses of Bioinformatics	B3, C3, F3	
Communication in Bioinformatics	K3	
Continuum Development in Bioinformatics	L3, M3	Affective

### 2.2 Domain topics—refining ISCB core competencies

A major update in Core Competencies 3.0 involves the inclusion of KSAs that collectively define competency fulfillment. Additionally, two new topics were incorporated: (i) Data Science, focused on Artificial Intelligence and Machine Learning, and (ii) Professional Conduct and Career Development. Finally, multiple competencies underwent categorical shifts from their Core Competencies 2.0 classifications. All versions remain accessible via the Competency Hub (https://competency.ebi.ac.uk/framework/iscb/3.0/competencies).

Our classification of Core Competencies 3.0 builds upon the domain framework previously established by RSG-Brazil’s Educational Committee (Table 1, available as supplementary data at *Bioinformatics Advances* online), integrating updates from version 3.0 (Brooksbank *et al.* 2024). For each topic, we systematically evaluated all KSAs to determine their alignment with prior classifications. This assessment guided categorization decisions: maintenance within existing domains, updated classification within domains, or creating new domains where necessary.

### 2.3 Survey design, research development, and distribution

To assess the needs of Brazil’s bioinformatics community, we designed a Google Forms survey collecting: (i) consent compliant with Brazil’s General Personal Data Protection Law (Lei Geral de Proteção de Dados Pessoais, LGPD, Law No. 13 709/2018); (ii) ethnic/racial self-declaration; (iii) state; (iv) academic information; (v) gender identification; (vi) educational background; (vii) specialization area; and (viii) professional sector (academia/industry). Participants also self-classified as bioinformatics users, scientists, or developers, aligning with Competency Hub categories (https://competency.ebi.ac.uk/). Survey themes mirrored key topics from ISMB (ISCB Main Conference) and X-meeting (AB³C Conference): (i) DNA and Genomics; (ii) Phylogeny and Evolution; (iii) Metagenomics and Microbiome; (iv) RNA and Transcriptomics; (v) Epigenomics; (vi) Proteins and Proteomics; (vii) Metabolomics and System Biology; (viii) Artificial Intelligence and Data-Science; (ix) Databases and Software Development; (x) Molecular Bases of Cancer; (xi) Personalized Medicine; (xii) Scientific Communication; (xiii) Structural Biology and Modeling; and (xiv) Career Mentorship in Bioinformatics. The survey was disseminated via institutional email lists, social media, academic groups, and corporate networks.

### 2.4 Data collection and analysis

The survey remained accessible for 6 months; all questions are cataloged in Table 2, available as supplementary data at *Bioinformatics Advances* online. Upon closure, data were analyzed using R. The complete questionnaire, response dataset, and reproducible R analysis scripts, with all necessary dependencies, are publicly available in the RSG-Brazil EduCom GitHub repository (https://github.com/CursosRSG/Article_2025.git).

### 2.5 Ethical considerations and data protection

In compliance with Brazil’s General Personal Data Protection Law (Lei Geral de Proteção de Dados Pessoais, LGPD, Law No. 13 709/2018) and relevant regulations, all collected data were anonymized and used exclusively for this research. Personal identifiers were removed during analysis to prevent participant re-identification. Participation was voluntary, with participants retaining the right to withdraw at any time. Submission of responses constituted consent for the use of anonymized data as described. This study involves non-sensitive, anonymized online survey data and did not require formal ethics committee approval or written informed consent. All data are stored in password-protected RSG-Brazil drives with restricted access to authorized personnel, minimizing risks while ensuring privacy. No direct benefits were promised, though results aim to benefit Brazil’s bioinformatics community.

## 3 Results

### 3.1 Classification of core competencies into seven training domains

**Figure 1:**
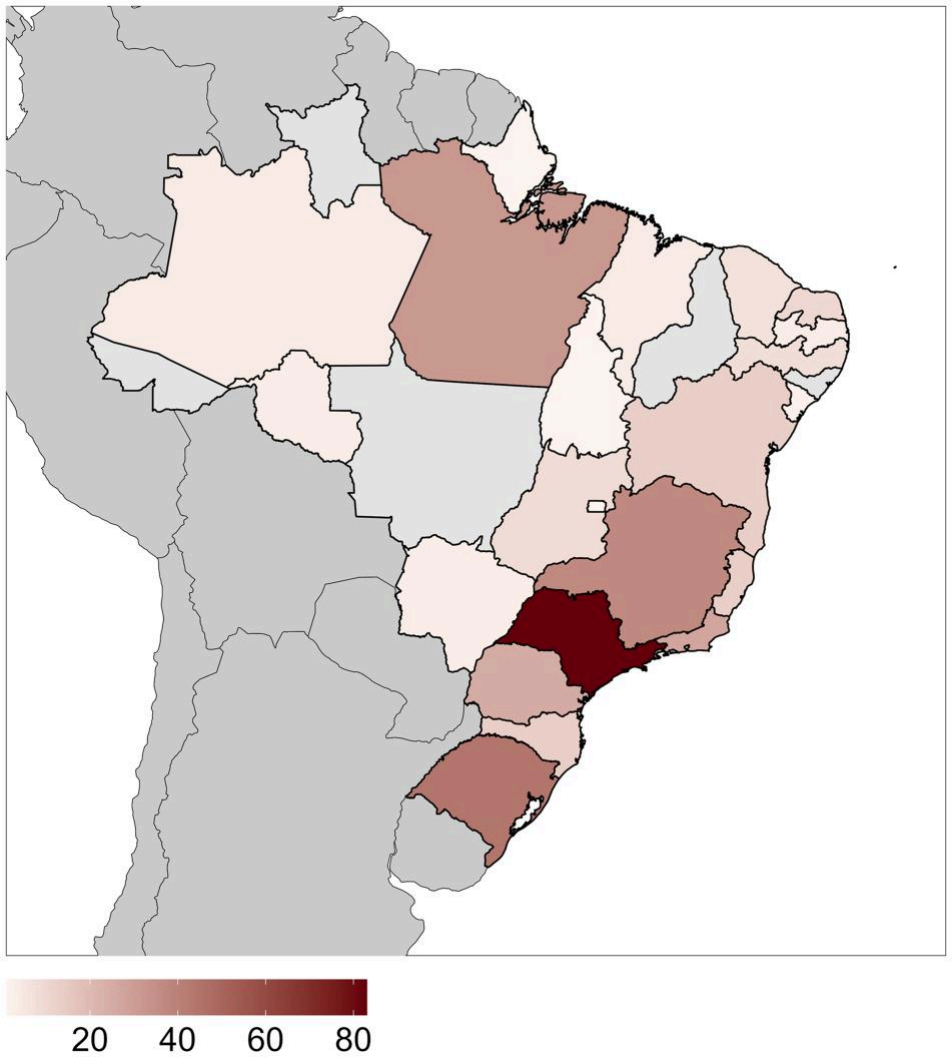
Geographic distribution of survey participants: response count per Brazilian state.

To systematize this classification, the RSG-Brazil Educational Committee established seven bioinformatics Education and Training domains (Table 1), aligning all Core Competencies 3.0 through refined KSA analysis. Survey responses totaled 375; after data cleaning, 362 were analyzed (see Section 2), covering all five Brazilian regions and 20 of 27 states (Fig. 1). Furthermore, participants were asked to classify themselves into three distinct profiles: (i) Users, who exclusively apply bioinformatics tools in research without developing them; (ii) Scientists, who both develop and apply bioinformatics tools in research; (iii) Developers, who focus exclusively on tool development without research application. The ISCB Core Competency Framework 3.0 is organized into topics (A3 to M3), each containing subtopics defined as KSAs that detail specific competencies. Topic A3, focusing on foundational biological concepts and skills, resides within the “Knowledge in Biology” domain. Topics B3, C3, and F3 are grouped under the “Uses of Bioinformatics” domain, reflecting their shared emphasis on competencies related to applying omics technologies. For example, KB3-1 describes the use of omics-scale and big-data-driven core life science technologies. SC3-2 addresses methodologies for ensuring data quality, while AF3-1 emphasizes the necessary skills for utilizing public biological data repositories.

The D3 topic is mapped to the “Methods in Statistics and Data Science” domain due to its subtopics (KD3-1, KD3-2, KD3-4, KD3-5), emphasizing the application of statistical and mathematical knowledge in bioinformatics. Subsequent KSAs further reinforce competencies in data analysis and the usage of statistical tests. Topics G3, H3, and I3 reside within the “Knowledge of Computing and Interdisciplinary Programming” domain, as their KSAs collectively address foundational computing skills: basic computing and scripting languages (KH3-1; SH3-1), website/GUI development (SG3-2), pipeline construction (SH3-6), software testing/debugging (SH3-4), human-computer interaction design (SG3-3), and system benchmarking for computational time estimation (SI3-4). The update from Core Competencies 2.0 to 3.0 introduced two new domains, “Communication in Bioinformatics” and “Continuum Development in Bioinformatics,” to address evolving professional requirements. Topic K3 resides within the “Communication” domain due to its emphasis on essential skills for engaging stakeholders beyond the field, formally establishing communication as an independent competency area. Concurrently, topics E3 and J3 are classified within the 'Ethical Implications and Repercussions of Bioinformatics in Society’ domain, as all their associated KSAs address broader societal and ethical considerations; examples include KE3-5 (responsible handling of sensitive data) and KJ3-4 (professional codes of conduct and publication ethics). Notably, E3 represents a novel competency in version 3.0, specifically emphasizing data management proficiency. Furthermore, bioinformaticians must evaluate their professional growth and identify collaborative needs, as positioning bioinformatics as a discipline requires continuous development. Topics L3 and M3 were mapped to the “Continuum Development in Bioinformatics” domain. Their KSAs include collaborative team dynamics (KL3-2), mentoring for knowledge transfer, and professional progression (SL3-2), which align predominantly with Bloom’s affective domain, which targets the development of attitudes, values, ethical commitments, and motivation through hierarchical progression from awareness to behavioral internalization, in contrast with the cognitive domain focus on intellectual skill acquisition.

### 3.2 Geographic and academic distribution

Bioinformaticians in Brazil exhibit concentrated distribution, with São Paulo, Minas Gerais, Rio de Janeiro, and Rio Grande do Sul serving as key hubs—a pattern reflecting these states’ advanced academic infrastructure, including major postgraduate programs and specialized enterprises in bioinformatics. Nationwide academic representation spans 16 states with master’s degree holders, 18 doctoral researchers, 15 postdoctoral scholars, and 9 adjunct professors. Despite this national coverage, five states (Roraima, Acre, Mato Grosso, Piauí, and Alagoas) remain unrepresented in our survey (Fig. 1, available as supplementary data at *Bioinformatics Advances* online).

### 3.3 Professional profiles and demographics

**Figure 2:**
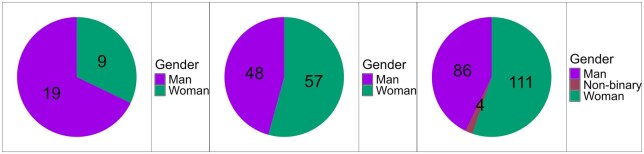
Gender distribution across User, Scientist, and Developer profiles in the survey.

Survey participants first self-reported their gender identity. Both the User and Scientist groups showed female predominance, whereas Developers were predominantly male. No transgender participants (female or male) were recorded, with four non-binary individuals exclusively within the User profile (Fig. 2). Participants subsequently self-classified as Users, Scientists, or Developers, revealing distinct professional distributions and needs. Most are identified as Users (predominantly academics in undergraduate/graduate programs), followed by Scientists and Developers.

**Figure 3:**
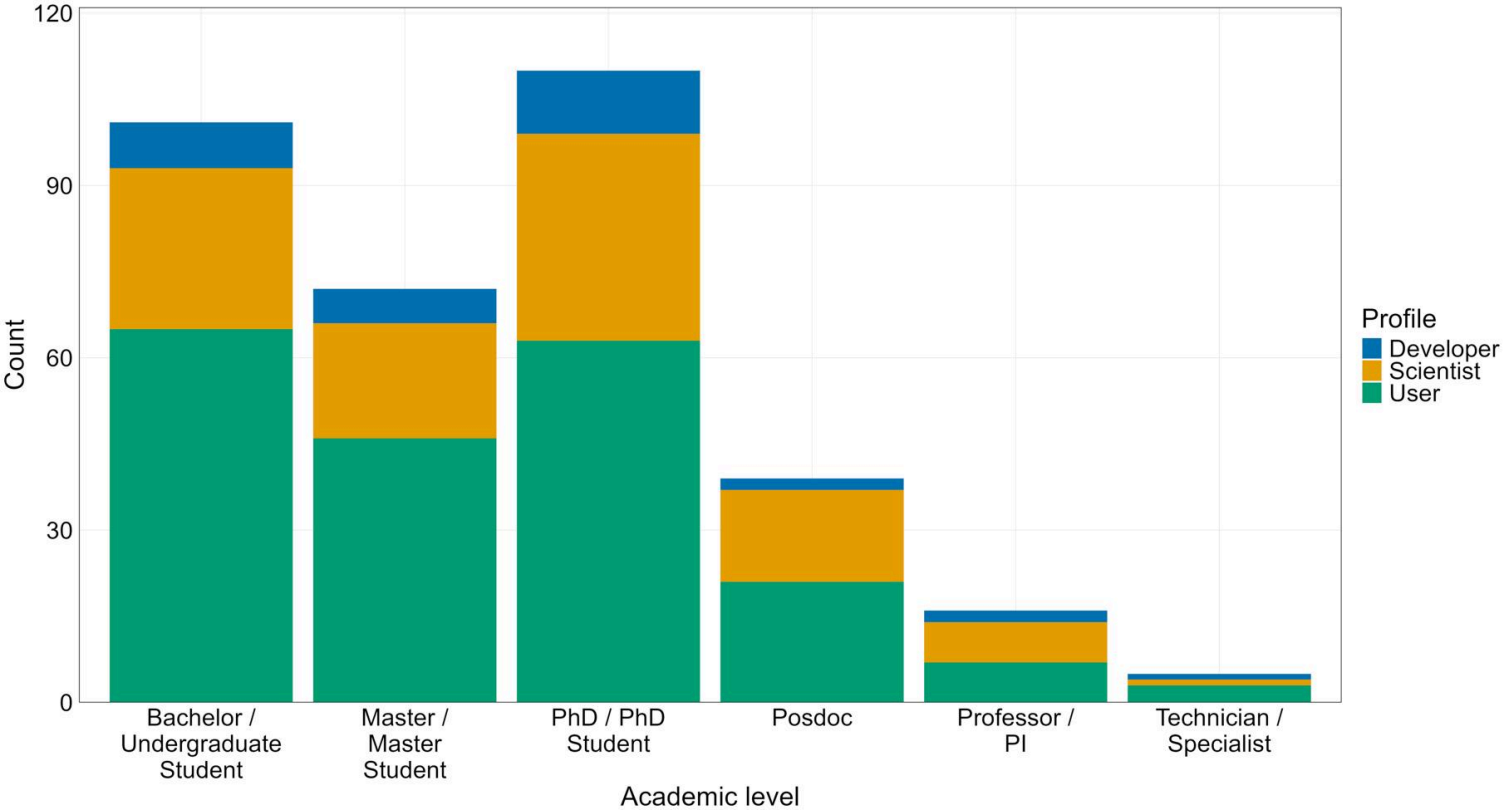
Education level distribution across professional profiles: User (green), Scientist (yellow), and Developer (blue).

The Scientist profile demonstrated the broadest role diversity, spanning diverse subdisciplines (Fig. 3). Survey respondents reflect the demographic diversity of Brazilian bioinformaticians, with most identifying as Users, followed by Scientists and Developers. The diverse academic backgrounds of participants, spanning undergraduates, master’s candidates, doctoral researchers (PhDs), postdoctoral scholars, technicians, and professors, demonstrate the multidisciplinary nature of bioinformatics (Fig. 3). Educational attainment differs significantly across profiles: Developers and Scientists predominantly hold master’s or doctoral degrees, while Users are primarily undergraduates. Professional roles include clinicians, biocurators, professors, and ethics specialists. Scientists exhibit particular heterogeneity across subfields: computer scientists, core facility researchers, computational biology educators, and biomedical informaticians (Fig. 3, available as supplementary data at *Bioinformatics Advances* online). This comprehensive profile analysis enables targeted development of bioinformatics training programs through curricula aligned with community needs.

### 3.4 Confidence levels and training demands

To develop targeted educational programs, we analyzed participant confidence levels across the seven ISCB Core Competencies 3.0 domains by career profile (Fig 2, available as supplementary data at *Bioinformatics Advances* online). This mapping elucidates educational needs within Brazil’s bioinformatics community, using a 7-point scale (1 = least confident, 7 = most confident). The “Knowledge in Biology” domain showed diverse confidence patterns: scientists and users peaked at 7, while developers peaked at 5. For “Methods in Statistics and Data Science,” all profiles peaked at 7, with developers reporting no scores at 2 or 4. Scientist and user profiles consistently exhibited ascending confidence distributions across multiple domains. This pattern recurred in “Knowledge of Computing and Interdisciplinary Programming,” “Uses of Bioinformatics,” and “Continuous Development in Bioinformatics,” where all profiles showed majority responses at 7, and increasing frequency toward higher confidence levels.

The “Ethical Implications and Repercussions of Bioinformatics in Society” domain exhibited relatively uniform confidence distributions across all profiles, with most scores showing comparable response frequencies. The sole exception was the lowest confidence value (1), which had the fewest responses in all profiles. Conversely, the “Communication in Bioinformatics” domain revealed distinct profile-based patterns: Users strongly emphasized its importance, while Developers showed minimal representation**—**aligning with their primary focus on technical development. Scientists, being predominantly academic, indicated that communication is a skill that requires curricular development.

**Figure 4:**
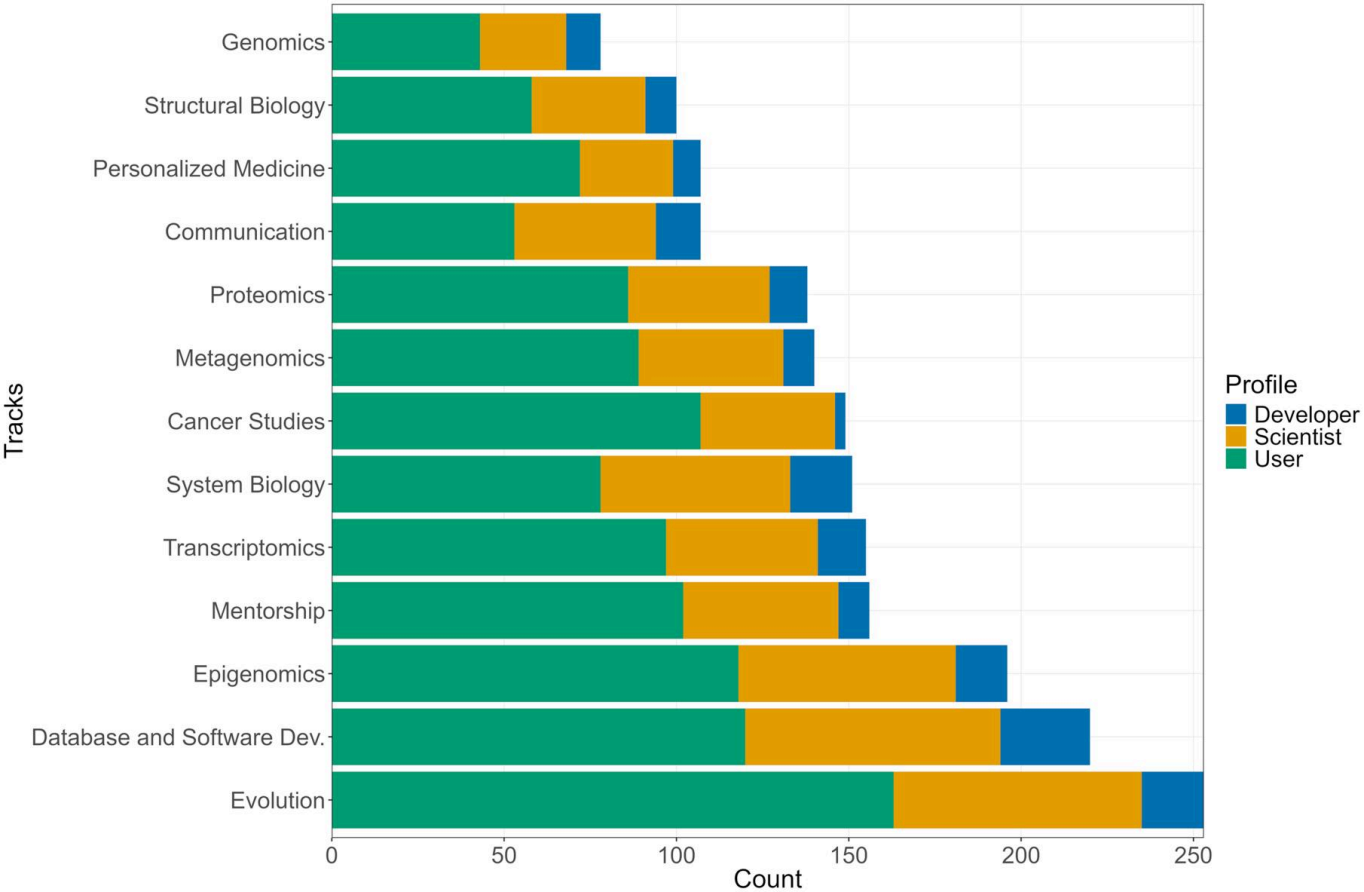
Training demand among survey participants across User, Scientist, and Developer profiles.

**Figure 5:**
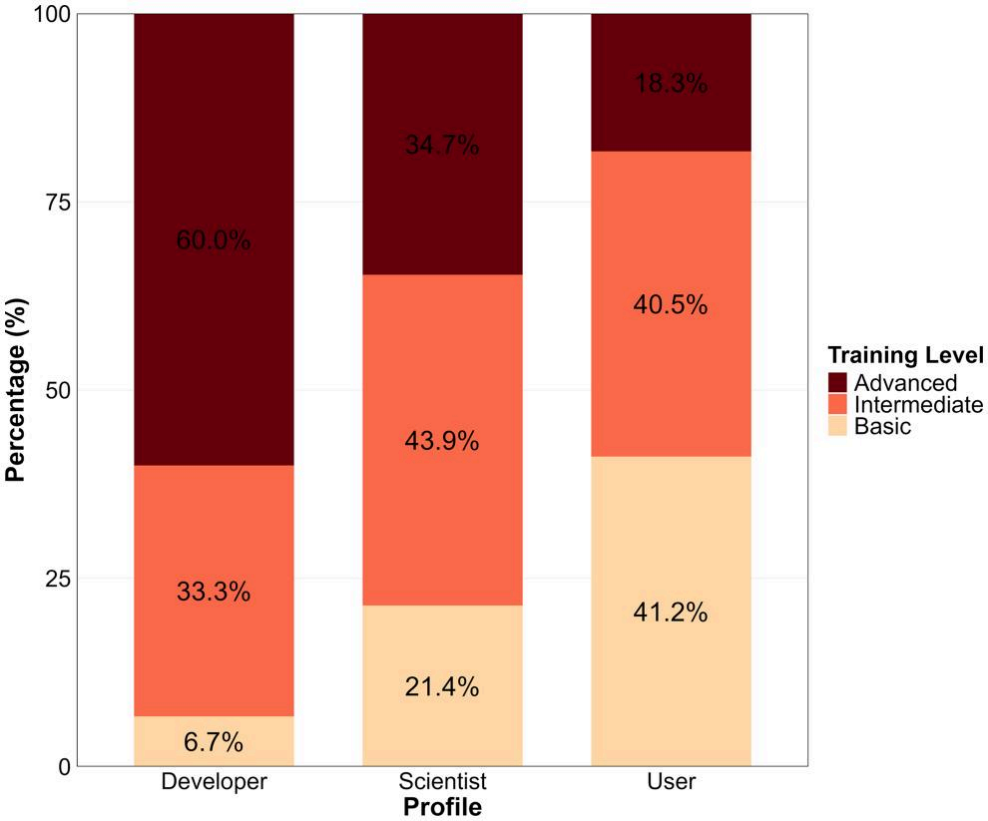
Preferred course complexity levels among survey participants across User, Scientist, and Developer profiles.

Table 1 summarizes these seven essential domains for bioinformaticians: (i) Knowledge in Biology, (ii) Methods in Statistics and Data Science, (iii) Knowledge of Computing and Interdisciplinary Programming, (iv) Ethical Implications and Repercussions of Bioinformatics in Society, (v) Uses of Bioinformatics, (vi) Communication in Bioinformatics, and (vii) Continuous Development in Bioinformatics, providing a vital framework for educational planning. Participants indicated demand for specialized bioinformatics courses through a multiple-response question featuring common study areas (Fig. 4). Phylogenetics/evolution courses were the most requested overall and represented the top choice among Users. Database and software development courses ranked second overall, emerging as the primary preference for both Developers and Scientists. Epigenomics placed third overall. Notably, systems biology received the fewest votes (∼90) across all profiles. These findings guide RSG-Brazil’s Educational Committee in prioritizing community-relevant course development. Training level requirements also differed substantially:

Users predominantly need basic/intermediate courses with minimal advanced training, whereas Scientists require advanced/intermediate content with limited basic instruction. Developers show a dual pattern, primarily requesting advanced courses despite indicating baseline competency needs (Fig. 5). Industry professionals constituted 18% of survey respondents (primarily Developers and Scientists), revealing distinct training priorities critical for workforce readiness. These participants demonstrated significantly higher demand for database/software development courses with an emphasis on advanced competencies in cloud computing, AI integration, and scalable pipeline deployment, reflecting commercial requirements for production-grade bioinformatics solutions in diagnostics, agricultural biotechnology, and therapeutic development. This comprehensive 6-month survey (November 2023–May 2024) engaged Brazil’s scientific community to assess bioinformatics education and training. Disseminated through emails, specialized groups, websites, and social media, it reached bioinformaticians across all major regions. Results reveal pronounced geographic disparities: students and professionals cluster predominantly in São Paulo, Minas Gerais, and Rio Grande do Sul (South/Southeast), while Central-West, Northeast, and North regions show notably sparse representation.

## 4 Discussion

This imbalance highlights the uneven distribution of bioinformatics resources and educational opportunities. Bioinformatics inherently integrates exact and biological sciences, driving continuous technological innovation in biological research. Consequently, professional training must evolve in parallel with these conceptual and technical advancements. The foundational step in bioinformatics education is identifying professional training needs, requiring collaborative input from diverse experts capable of: (i) offering multifaceted perspectives, (ii) fostering skill-development collaborations, and (iii) pinpointing training gaps. Currently, Brazil lacks defined career pathways in bioinformatics, necessitating systematic studies to identify role-specific training requirements. Bioinformatics content remains scarce within undergraduate curricula across health, natural, and exact sciences. The field’s interdisciplinary nature demands comprehensive curricular reform in these programs. Although national initiatives have sought to categorize and expand Brazilian bioinformatics (Rocha *et al.* 2022), the need persists for a comprehensive assessment of bioinformatics curricula across undergraduate and graduate programs. As Latin America’s largest nation, Brazil contributes to more than 50% of the region’s scientific output and maintains a significant global research impact (Barata *et al.* 2018). However, multiple Brazilian states demonstrate minimal bioinformatics engagement. Our geographical distribution of scientists aligns with Rocha *et al.* (2022), confirming regional disparities. Such spatial analysis enables tracking field expansion across career trajectories and regions. Given Brazil’s territorial expanse and sociocultural diversity, this study identifies Northern/Northeastern states where strategic educational enhancements and scientific collaboration could advance bioinformatics research. This development would support nature-based economies and establish emerging knowledge hubs in these regions.

Bioinformatics education in Brazil faces profound structural barriers that perpetuate regional disparities. Linguistic exclusion arises from the scarcity of Portuguese-language materials and English-dominant technical databases, disproportionately disadvantaging institutions with lower English proficiency, particularly in the North and Northeast, which host only 15% of the nation’s Master’s and PhD programs (Farias and Lima 2022). Infrastructural deficits exacerbate this gap: unequal access to computational hardware, stable internet, and reliable electricity impedes large-scale analyses in these regions, compounded by Brazil’s dependence on international equipment (Rocha *et al.* 2022). Resource hyperconcentration in the South-Southeast fuels a vicious cycle of faculty shortages and brain drain, as limited investment in peripheral regions accelerates talent migration abroad. Meanwhile, pedagogical fragmentation confines bioinformatics training predominantly to genetics courses, neglecting interdisciplinary integration essential for modern biosciences (Fukuto and Viboud 2024).

Brazil’s bioinformatics education reflects broader socioeconomic inequities, demanding systemic interventions across four critical axes: (i) infrastructure redistribution through public investment in high-performance computing resources across the North and Northeast regions; (ii) establishment of national Portuguese-language open educational resource repositories to democratize access to technical knowledge; (iii) faculty incentive programs combating brain drain via targeted grants and regional research hubs; and (iv) curricular reform embedding bioinformatics across biological and computational disciplines. Without these measures, bioinformatics will remain confined to Brazil’s academic elite, impeding advances in precision medicine, public health, and biotechnology.

Discussions on diversity in bioinformatics training highlight the need for a balanced combination of biological and technological proficiency. Professionals capable of both generating and interpreting data are essential for advancement, necessitating integrated educational approaches that ensure equitable access across diverse backgrounds and regions. Bioinformatics fundamentally comprises two core components: (i) a deep understanding of biological principles, and (ii) computational/programming literacy. Promoting equity, therefore, demands curricula delivering balanced training in both domains. Prioritizing bioinformatics education extends beyond material resources to cultivating inclusive learning environments where diverse academic backgrounds thrive, achieved by eliminating stereotypes and demonstrating the field’s accessibility. Enhanced heterogeneity enriches research while maintaining accessibility. Finally, inclusion encompasses knowledge access: bioinformatics requires computers and internet connectivity, yet equipment scarcity disproportionately affects interested individuals in low- and middle-income countries.

Mulder *et al.* (2018) established foundational bioinformatics competencies in Core Competencies 2.0, identifying essential topics spanning: general/evolutionary biology, statistics and data science methods, societal ethics, and effective communication. The Core Competencies 3.0 framework subsequently restructured and updated these into integrated categories (Table S1), merging and reclassifying competencies to address evolving community needs. Key enhancements prioritize ethical standards, professional competencies, and ongoing development. This progression demonstrates how bioinformatics dynamic evolution and the imperative for adaptive educational frameworks that equip professionals for emerging challenges (Pevzner and Shamir 2009).

While Brazil serves as the focal context, this study establishes a replicable framework for addressing bioinformatics education gaps across the Global South, positioning Brazil as an exemplar middle-income nation facing structural barriers common to resource-constrained regions. As the first national implementation of the ISCB Core Competencies 3.0, our seven-domain methodology (encompassing domain adaptation, profile-based needs assessment, and structural barrier identification) provides a transferable model for systemic educational reform. The linguistic exclusion, infrastructural deficits, and regional disparities documented here reflect challenges widely found in other Latin American countries. To catalyze broader applicability, we propose leveraging ISCB’s global networks to initiate systematic comparative studies across Latin American Regional Student Groups. Such collaborations would quantify shared priorities (e.g. Portuguese/Spanish-language bioinformatics content) while adapting interventions to local contexts, thereby establishing a replicable framework for evidence-based educational reform in resource-constrained settings worldwide.

This work represents the first national survey of bioinformatics research based on the Core Competencies 3.0 framework, providing valuable insights into researcher demographics (race, gender, and geographical location) and identifying critical gaps essential for formulating a national strategy for bioinformatics education. As Brazil’s bioinformatics education programs mature, efforts increasingly target molecular biology and genetics research. Parallelly, data analysis skills have become curriculum priorities, ensuring students grasp foundational principles of biological research. Bioinformatics now systematically integrates advanced statistical and computational methodologies for large-scale data analysis/aligning with ISCB’s emphasis on these approaches and preparing students for data-driven science. Brazilian programs embed these competencies, equipping graduates to develop computational solutions for complex problems. This focus on algorithmic and programming proficiency ensures graduates advance the field. However, survey responses reveal persistent demands for broader course offerings and reflect stark regional disparities in scientific development. When implementing data-driven curricula, it is crucial to include regions where even fundamental bioinformatics remains underdeveloped.

## Supplementary Material

vbaf311_Supplementary_Data

## Data Availability

The data underlying this article, including the survey instrument, anonymized response dataset, and analysis R script, are publicly available in the RSG-Brazil EduCom GitHub repository (https://github.com/CursosRSG/Article_2025.git).
