# Supplementary material for: Mapping educational needs in bioinformatics in Brazil: adapting ISCB 3.0 competencies to a regional context
Source: Bioinform Adv. 2025 Dec 2;5(1):vbaf311. doi: 10.1093/bioadv/vbaf311 (PMC12714386; doi:10.1093/bioadv/vbaf311)
Supplement: vbaf311_Supplementary_Data [file vbaf311_supplementary_data.docx]

# ***-Supplementary information-***

#

# **Mapping Educational Needs in Bioinformatics in Brazil: Adapting ISCB 3.0 2 Competencies to a Regional Context**

Bernardo Velozo^1^, Clara Carvalho^2^, Rayssa Feitosa^3^, Lucas Aleixo Leal Pedroza^4^, Emerson Danzer^5^, Sandy Ingrid Aguiar Alves^6^ , Maira Neves^2^ and Bibiana Fam^7*^

^1^ Programa de Pós-Graduação em Bioquímica, Departamento de Bioquímica, Instituto de Química, Universidade Federal do Rio de Janeiro, Rio de Janeiro, Brazil; ^2^ Programa Interunidades de Pós-graduação em Bioinformática, Universidade de São Paulo, São Paulo, Brazil; ^3^ The Hospital for Sick Children, Genetics and Genome Biology Department, Toronto, Canada; ^4^ Programa de Pós-graduação em Biologia Aplicada à Saúde, Instituto Keizo Asami, Universidade Federal de Pernambuco, Recife, Brazil; ^5^ Programa Interunidades de Pós-graduação em Bioinformática, Universidade Federal de Minas Gerais, Belo Horizonte, Minas Gerais, Brazil; ^6^ Programa de Pós-Graduação em Biologia de Agentes Infecciosos e Parasitários, Universidade Federal do Pará, Belém, Pará, Brazil; ^7^ Laboratory of Genomic Medicine, Center for Experimental Research, Hospital de Clínicas de Porto Alegre, Porto Alegre, RS, Brazil.

***Corresponding author:**

Bibiana Fam, Laboratory of Genomic Medicine, Experimental Research Center, Hospital de Clínicas de Porto Alegre. R. Ramiro Barcelos, 2350 - 21506, Porto Alegre, RS, Brasil. ORCID 0000-0002-6654-6415, [bsfam@hcpa.edu.br](mailto:bsfam@hcpa.edu.br)

**Table S1.** EduComm classification framework mapping ISCB Core Competencies 3.0 to domain-specific bioinformatics education areas.

| **ID*** | **Label** | **Competency** |
| --- | --- | --- |
| **1** | A | General Biology. |
|  | B | Depth in at least one area of biology (e.g., evolutionary biology, genetics, molecular biology, biochemistry, anatomy, physiology). |
|  | C | Biological data generation technologies. |
|  | D | Details of the scientific discovery process and of the role of bioinformatics in it. |
| **2** | E | Statistical research methods in the context of molecular biology, genomics, medical and population genetics research. |
| **5** | F | Bioinformatics tools and their usage. |
| **3** | G | The ability of a computer-based system, process, algorithm, component or program to meet desired needs in scientific environments/problems. |
|  | H | Computing requirements appropriate to solve a given scientific problem (e.g., system, process, algorithm, component, or program; define algorithmic time and space complexities and hardware resources required to solve a problem). |
|  | I | GUI/Web-based computing skills appropriate to the discipline (e.g., effectively use bioinformatics and analysis tools through the web). |
|  | J | Command line and scripting-based computing skills appropriate to the discipline. |
|  | K | Construction of software systems of varying complexity based on design and development principles. |
| **4** | L | Local and global impact of bioinformatics and genomics on individuals, organizations, and society. |
|  | M | Professional, ethical, legal, security, and social issues, and responsibilities of bioinformatics and genomic data in the workplace. |
| **5** | N | Effective communication of bioinformatics and genomics problem/issue/topics with a range of audiences, including, but not limited to, other bioinformatics professionals. |
|  | O | Effective teamwork to accomplish a common scientific goal. |
|  | P | Engage in continuing professional development in bioinformatics. |

* 1, Knowledge in Biology; 2, Methods in Statistics; 3, Knowledge in Computation and Programming;

4 - Ethical Implications and Bioinformatics Repercussions, and 5 - Uses of Bioinformatics and

Communication

**Table S2.** Complete survey instrument administered to study participants.

| **ID** | **Question** |
| --- | --- |
| 1 | I declare that I have read, understood, and wish to participate in the survey conducted by the ISCB Regional Student Group - Brazil. |
| 2 | Race/Color? |
| 3 | Which of the following terms do you think best describes your gender? |
| 4 | In which state of Brazil do you currently work/study/live? |
| 5 | What is your academic level? |
| 6 | According to your experience, which of the options best describes you? |
| 8 | Among these options, which one best describes your professional profile? |
| 9 | Among these options, which one best describes your professional profile? |
| 10 | Taking into account your needs, which aspects of Bioinformatics would you most like to see in a course? [Biology knowledge] |
| 11 | Considering your needs, which aspects of Bioinformatics would you most like to see in a course? [Statistical Methods and Data Science] |
| 12 | Considering your needs, which aspects of Bioinformatics would you most like to see in a course? [Computational and programming skills] |
| 13 | Considering your needs, which aspects of Bioinformatics would you most like to see in a course? [Ethical implications and societal repercussions of Bioinformatics] |
| 14 | Considering your needs, which aspects of Bioinformatics would you most like to see in a course? [Applications of Bioinformatics] |
| 15 | Considering your needs, which aspects of Bioinformatics would you most like to see in a course? [Communication in Bioinformatics] |
| 16 | Considering your needs, which aspects of Bioinformatics would you most like to see in a course? [Continuing Development in Bioinformatics] |
| 17 | Among the topics below, which one(s) would you like to deepen your knowledge and develop skills in Bioinformatics? |
| 18 | Considering your experience in the field, at what level of learning would you like to receive courses and training offered in Computational Biology? |


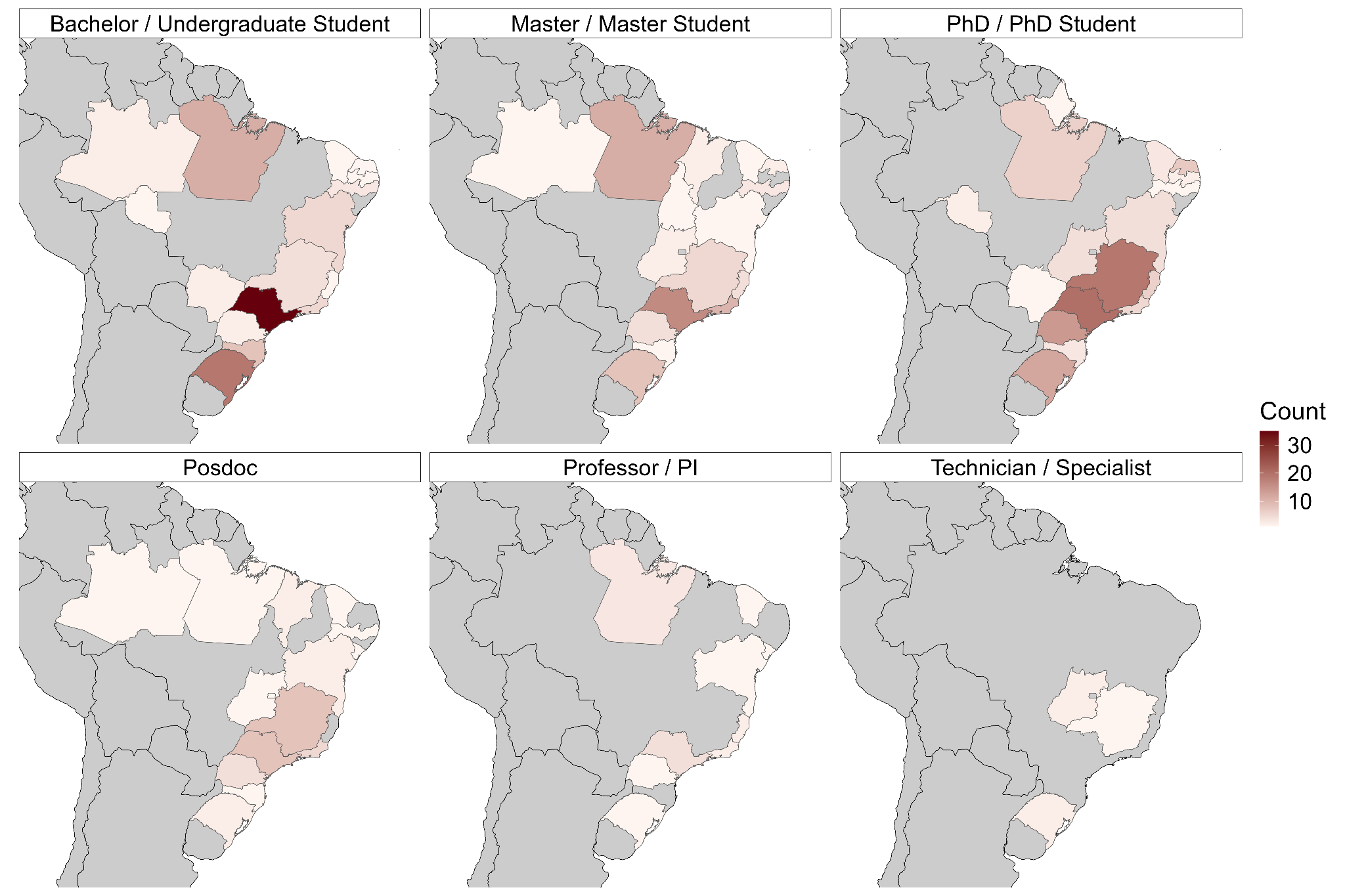


**Figure S1.**  Geographic distribution and academic attainment levels of survey participants across Brazilian states.

**
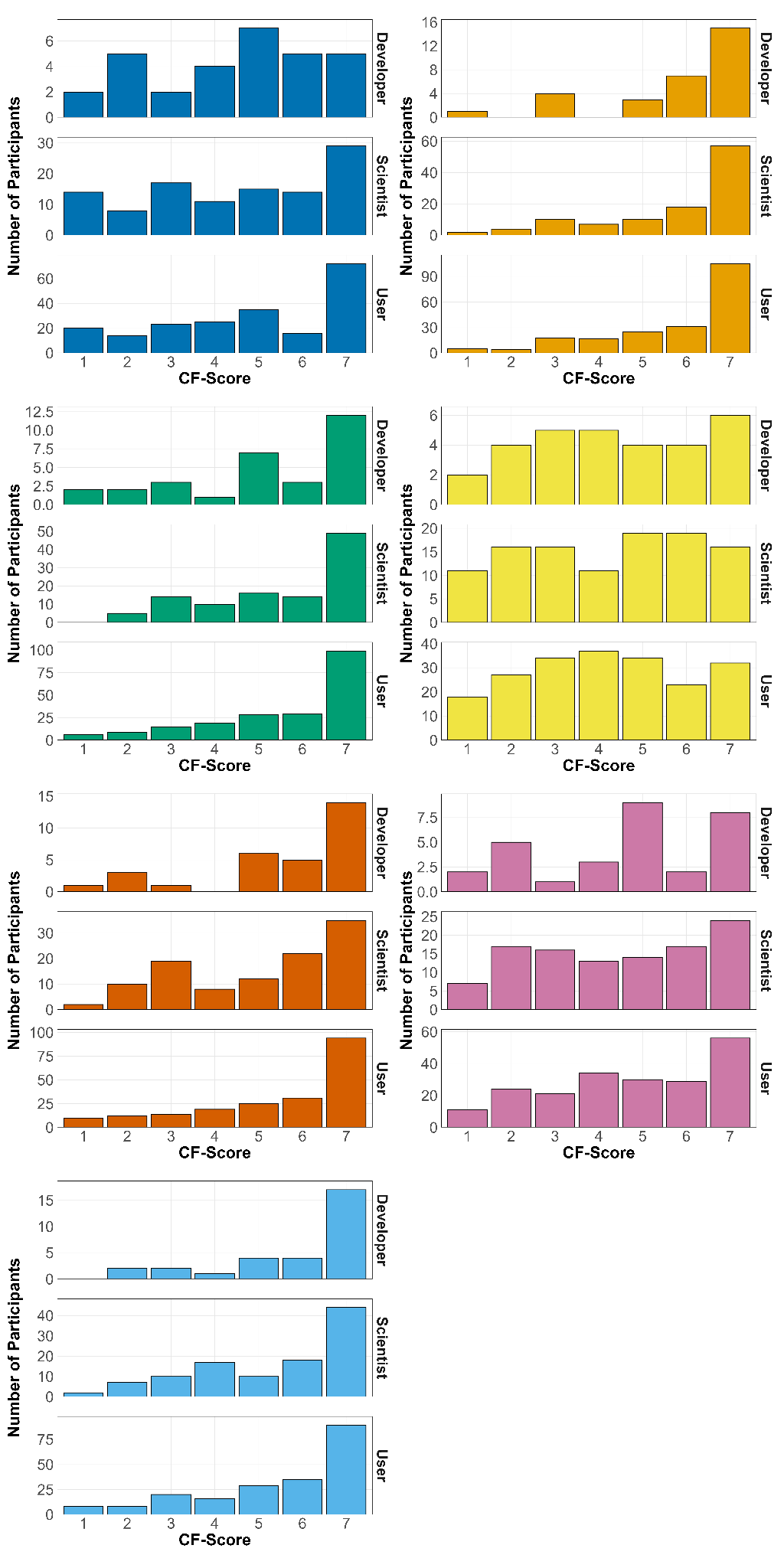
**

**Figure S2.** Confidence ratings across ISCB Core Competencies 3.0 domains by professional profile: User, Scientist, and Developer.


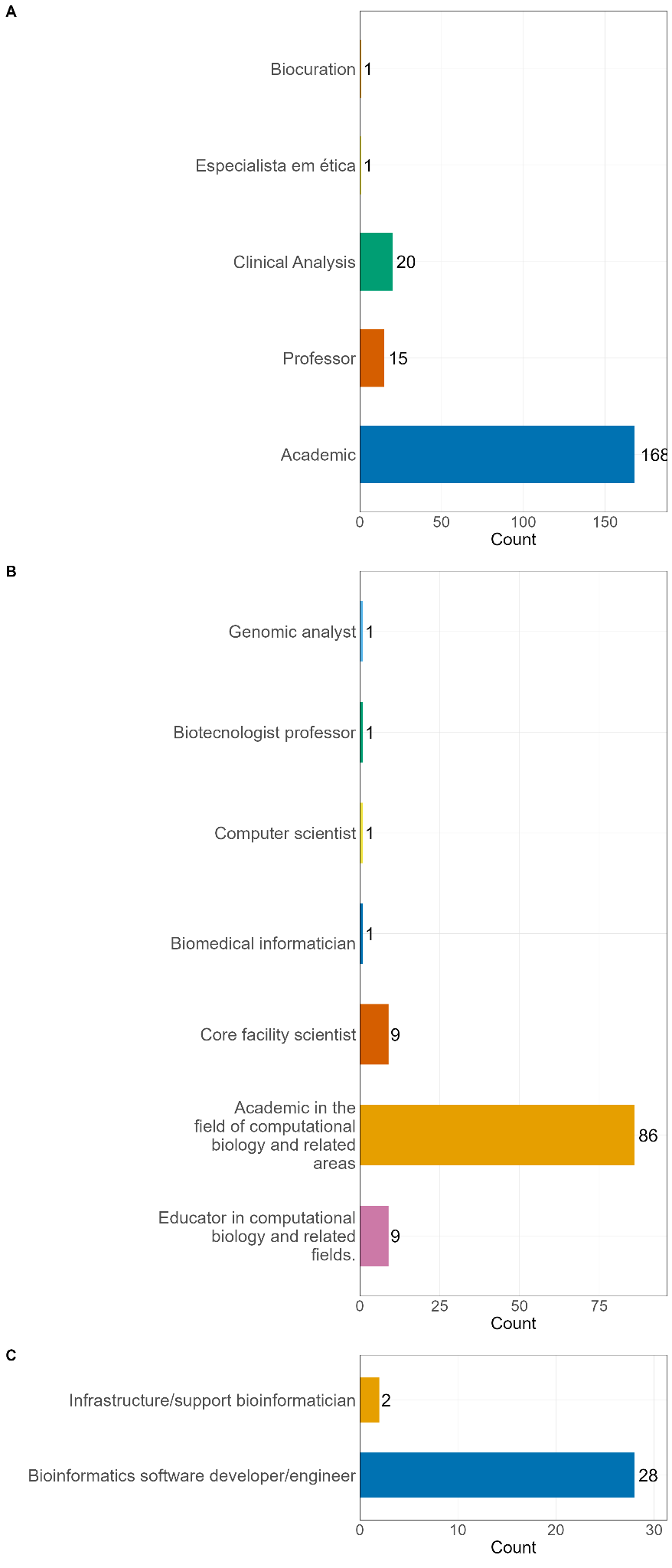


**Figure S3.** Career role distribution across professional profiles in the Brazilian bioinformatics community: User, Scientist, and Developer.
